# Deconstruction of Lignin: From Enzymes to Microorganisms

**DOI:** 10.3390/molecules26082299

**Published:** 2021-04-15

**Authors:** Jéssica P. Silva, Alonso R. P. Ticona, Pedro R. V. Hamann, Betania F. Quirino, Eliane F. Noronha

**Affiliations:** 1 Enzymology Laboratory, Cell Biology Department, University of Brasilia, 70910-900 Brasília, Brazil; wpinheiro.jessica@gmail.com (J.P.S.); alonso.poma.t@gmail.com (A.R.P.T.); pedror_hamann@hotmail.com (P.R.V.H.); 2 Genetics and Biotechnology Laboratory, Embrapa-Agroenergy, 70770-901 Brasília, Brazil; betania.quirino@embrapa.br

**Keywords:** lignin, bacteria, biodegradation, auxiliary activities, metagenomics, metaproteomics, metatranscriptomics

## Abstract

Lignocellulosic residues are low-cost abundant feedstocks that can be used for industrial applications. However, their recalcitrance currently makes lignocellulose use limited. In natural environments, microbial communities can completely deconstruct lignocellulose by synergistic action of a set of enzymes and proteins. Microbial degradation of lignin by fungi, important lignin degraders in nature, has been intensively studied. More recently, bacteria have also been described as able to break down lignin, and to have a central role in recycling this plant polymer. Nevertheless, bacterial deconstruction of lignin has not been fully elucidated yet. Direct analysis of environmental samples using metagenomics, metatranscriptomics, and metaproteomics approaches is a powerful strategy to describe/discover enzymes, metabolic pathways, and microorganisms involved in lignin breakdown. Indeed, the use of these complementary techniques leads to a better understanding of the composition, function, and dynamics of microbial communities involved in lignin deconstruction. We focus on omics approaches and their contribution to the discovery of new enzymes and reactions that impact the development of lignin-based bioprocesses.

## 1. Introduction

The conversion of lignocellulosic biomass into biofuels and chemicals has gained interest because of its potential application in biorefineries as a green platform. Lignocellulosic biomass is mainly composed of lignin and polysaccharides (i.e., cellulose, hemicellulose, and pectin), arranged in plant cell walls. Cellulose is a homopolymer, composed of d-glucose monomers joined by linear β (1–4) linkages. Hemicellulose is also a sugar polymer, but it is composed of different monosaccharide molecules mainly joined by β-1,4 glycosidic linkages. Among the sugars that compose hemicellulose, d-xylose, d-mannose, and arabinose are present. Hemicellulose polymers are strongly interlinked through covalent and non-covalent bonds, and also linked to lignin, which together with cellulose will form the recalcitrant lignocellulosic matrix [1,2]. In the plant cell wall, lignin is linked to the carbohydrate moiety via the ester linkage. This association gives the plant cell wall greater strength and impermeability [3]. Lignin is formed by radical coupling reactions involving the three main phenylpropane units: p-hydroxyphenyl (H), guaiacyl (G), and syringyl (S), linked by C–C and C–O linkages [4,5].

Lignin is the main plant cell wall component responsible for recalcitrance [6]. Thus, pretreatment is an essential step for removing lignin in the process of lignocellulosic biomass conversion into biofuels [7,8]. The high carbon/oxygen ratio and the natural abundance of lignin make it a promising feedstock material for biological conversion into value-added products [9]. In addition to the biofuel industry, lignin may also be found as a by-product from wood-biomass in industrial processes for paper and pulp production. At least 75,000 tons of kraft-lignin were commercialized in 2018, and for the year 2025, 250,000 tons are expected [10].

The efficient degradation of lignin by white-rot basidiomycete fungi has been extensively studied [11,12]. Bacteria are also capable of deconstructing lignin [5,13], but they are less studied in comparison to fungi [12,14]. Recently, bacteria have been attracting great attention due to their high adaptability and biochemical versatility. Furthermore, bacteria have metabolic pathways that convert lignin and its derivates into products of biotechnological interest such as lactic acid, pyruvate, vanillin, lipids, polyhydroxyalkanoates (PHA), and *cis*-muconic acid (*cis*, *cis*-MA) [15,16].

In nature, lignin is deconstructed by the concerted efforts of microbial communities, rather than isolate microorganisms. Therefore, the strategy of studying the microbial community may provide a broader comprehension of the process of lignin deconstruction. In natural environments, different microorganisms in the microbial community operate synergistically through the secretion of a variety of biocatalysts. Although in nature cooperation between different microorganisms is common, in the laboratory, not all organisms are easily cultured, which is an obstacle to adapting the natural synergistic lignin deconstruction to its bioconversion in industrial processes. However, investigation of natural microbial communities using culture-independent approaches (e.g., metagenomics, metatranscriptomics, and metaproteomics) may deliver a detailed description of the processes and enzymes involved. Thus, the study of lignin-degrading microbiomes (e.g., forests, animals’ digestive tract, and sewage [17,18]) may be crucial to the development of efficient industrial processes for the use of ligninolytic feedstocks.

In the present review, we explore the contribution of omics approaches in discovering/describing new enzymes and microorganisms in microbiomes adapted to degrade lignin. Primarily, we focus on ligninolytic enzymes belonging to the auxiliary activities (AA) family, with emphasis on their impact on lignin valorization in industrial processes.

## 2. Lignin

### 2.1. Structure and Composition of Natural Lignin

**Figure 1:**
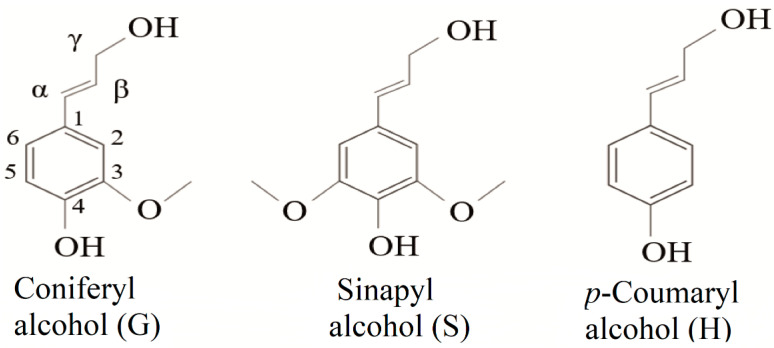
Chemical structures of the three monomeric precursors of the lignin macromolecule.

Lignin is a phenolic macromolecule of high molecular weight, composed of three main units of phenylpropane (monolignols): coniferyl alcohol, sinapyl alcohol, and *p*-coumaryl alcohol (Figure 1). When incorporated into the lignin structure, these monomers are termed *p*-hydroxyphenyl (H), guaiacyl (G), and syringyl (S) units, respectively [19,20,21]. In addition to these three main units, other subunits are present in the lignin structure. Among them are ferulic acid, ferulates, coniferaldehyde, synapaldehyde, 5-hydroxyiconiferyl alcohol, and acylated monolignols that contain acetate, *p*-coumarate [21,22,23].

Depending on its origin, lignin can be classified into three main groups: softwood, hardwood, and grass lignins. Softwood lignins are mainly composed of guaiacyl units, and thus are classified as G-type lignin. Hardwood lignins are mainly composed of different proportions of guaiacyl-syringil (GS-type) [24,25]. Grass lignins have a mixture of the three aromatic units (HGS), and contain a higher proportion of *p*-coumaryl alcohol (H)-derived units than other lignin types [26,27]. The highest proportion of lignin is found in softwoods (24–33%), while hardwoods and grasses have a smaller amount (~15–28%) [4,28].

**Figure 2:**
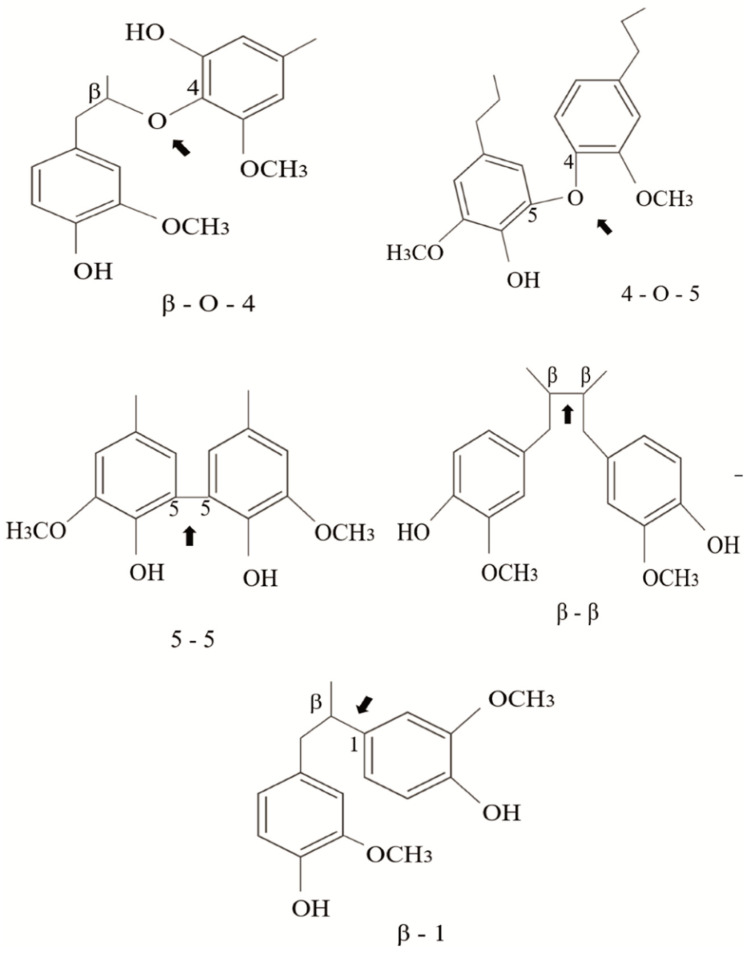
Typical linkages found in the structure of natural lignin; ether bonds (β-O-4′, 4-O-5′) and condensed bonds (5–5′, β-5′, β-β′, β-1′).

The linkages between phenylpropane units can be ether bonds (β-O-4′, 4-O-5′) or carbon–carbon bonds (5–5′, β-5′, β-β′, β-1′) usually called condensed bonds (Figure 2). The relative abundance of these linkages depends on the type of monomer present in the lignin structure. For example, G-type lignins contain more resistant linkages (β-5, 5-5) than S-rich lignins that are less condensed-bonded through ether bonds [23].

In the plant cell wall, lignin is covalently linked to the hemicellulose matrix, forming the lignin-carbohydrate complex (LCC). Among the types of linkages that are formed in the LCC, there are phenyl glycosides, benzyl ester, and ferulate or deferulate esters that are linked to lignin in 4-OH and 4-O positions [29,30]. Phenyl-glycoside bonds are one of the most common linkages in the LCC complex and are established between the carbohydrate reducing ends and the phenolic terminal groups of the lignin macromolecule. The benzyl ester linkages connect the lignin and carbohydrate moieties through the uronic acid of the carbohydrates and the hydroxyl group of the lignin. Ferulate or deferulate esters form most of the LCC linkages in grasses [31]. Ferulate mediates the crosslinks between polysaccharide–polysaccharide and polysaccharide–lignin. Crosslinks make the lignin physically close to the cell wall polysaccharides in such a way that lignin blocks the access of enzymes to the polysaccharides [19,26].

### 2.2. Technical Lignins

Technical lignins are produced as by-products in the pretreatment of lignocellulose procedures to obtain the carbohydrate moiety. These procedures include physical, chemical, and physicochemical methods, which result in a liquid residue known as black liquor, mainly composed of lignin, carbohydrates, and ash. Processing methods such as kraft, lignosulfonate, soda, organosolv, and hydrolyzed lignin are widely used, and each lignin produced is unique in terms of chemical structure, impurity profile, polydispersity, and molecular weight [32,33,34].

Technical lignin has been used for the development of products such as binders, dispersants or emulsifiers, and sequestrants. Almost 90% of commercial lignin products in the world are lignosulfonates, often used as polymeric anionic dispersants. Technical lignin has also been used for civil engineering applications such as soil stabilization, asphalt stabilization, and cement additives [35,36,37].

The kraft procedure is the primary pulping process and uses a mixture of sodium hydroxide (NaOH) and sodium sulfide (Na_2_S) solution at high temperatures (150–180 °C), which breaks ether bonds in lignin through an episulfide intermediate. During cooking, lignin is degraded into fragments of different molecular weights and is dissolved into the pulping liquor. After separating cellulose by filtration, the lignin and remaining hemicellulose in the liquid phase (black liquor) can be used in industrial processes. In modern pulp mills, the lignin present in black liquor is incinerated to supply energy for the mill. Isolation of lignin from black liquor for conversion into valuable products such as biofuel or a chemical compound might be an interesting alternative. Indeed, a lignin isolation procedure from black liquor known as lignoboost has been introduced. This procedure begins with adjusting the pH (up to 9.5) of the black liquor with the addition of carbon dioxide (CO_2_). In the next step, the recovered lignin is re-slurried using sulfuric acid (H_2_SO_4_) to pH 2, and finally, the lignin is isolated by filtration [38,39].

Kraft lignin shows different properties, distinguishing it from native lignin and other technical lignins. For instance, it has a significant increase of phenolic hydroxyl groups due to extensive cleavage of β-aryl bonds during the cooking process; furthermore, condensed structures and biphenyl are formed as a result of the severe procedure conditions. Lignin condensation usually occurs by the formation of new intermolecular C–C bonds between lignin fragments. Indeed, C–C bonds such as β–β and β–5 linkages are more difficult to disrupt than C–O bonds (β-aryl ether linkages). The degree of lignin condensation is important as it affects lignin recovery after biomass pretreatment. Preventing C–C bond formation during pretreatment reaction is an important positive variable for downstream utilization of lignin [30,34,40].

Lignosulfonates are obtained as by-products of sulfite cooking, in which delignification of biomass occurs by the addition of sulfite (SO_3_^2−^) and bisulfite (HSO_3_^−^). In this procedure, lignin is sulfonated, degraded, and solubilized. Lignosulfonates contain a large number of functional groups such as carboxylic, sulfur, and phenolic hydroxyl groups. Moreover, it presents distinctive physical and chemical characteristics such as solubility in water, high ash content, and relatively high molecular weight [40].

In the organosolv process, a mixture of organic solvent/solvents and water are added to the biomass, and these are essentially cooked at high temperatures (190 °C). Solvents such as ethanol, formic acid, acetic acid, and peroxiorganic acids are commonly used in this process. After this procedure, lignin is separated via solubilization. The lignin obtained by this process is less modified. Therefore, the main features of organosolv lignins are low molecular weight, high chemical purity, and the presence of many reactive side chains available for further chemical reactions, however, it shows poor solubility in water [38,39].

Depending on the conditions, and nature of the procedure that the lignocellulose biomass is subjected to, the resulting lignin might undergo irreversible repolymerization. In this case, lignin’s innate recalcitrance is enhanced, making it more challenging to produce value-added chemicals from it. In some cases, significant amounts of inhibitory products such as phenolic acids, furfurals, 5-hydroxymethylfurfural, and aldehydes are generated, and these can negatively impact downstream processes such as cellulose hydrolysis and fermentation [41].

Ionic liquids (ILs) are green solvents that can also be used for biomass pretreatment. In this case, dissolution of lignin contained in lignocellulosic biomasses is accomplished by using organic salts (i.e., alkylbenzenesulfonate, *N*-methylimidizole, and dimethylsulfoxide) that remain as liquids at relatively low temperatures (100 °C). Although ILs are not yet available at an industrial scale, there is growing interest in the utilization of ILs for the processing of biomass. IL pretreatments tend to preserve β-O-4 linkages in the lignin structure, maintaining similar properties to organosolv lignin. Lignin can be recovered from the ILs by precipitation via the addition of non-solvents such as water or acetonitrile [30,33].

## 3. Bacterial Enzymes for Lignin Deconstruction

**Figure 3:**
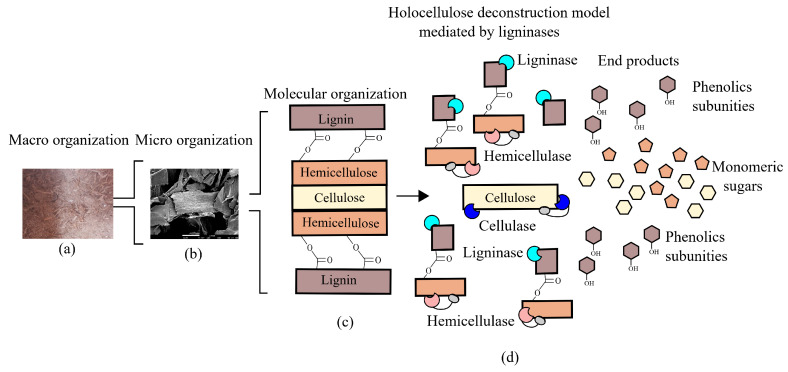
Cooperative model of lignin active enzymes and holocelullases in lignocellulosic biomass deconstruction. (**a**) Macro organization (sugarcane bagasse) and (**b**) micro organization (scanning electron microscopy) images are courtesy of Hamann P.R.V. (**c**) Carbohydrates and lignin organization in the plant cell wall. (**d**) cooperative activity of hemicellulases, cellulases, and ligninases to deconstruct lignocellulose.

Degradation and modification of lignin by bacteria involve a repertoire of redox-active enzymes classified in auxiliary activity (AA) families, according to the Carbohydrate-Active enZYmes (CAZy) Database [42]. These auxiliary activities involved in lignin breakdown primarily include the AA1 family, which contains laccases; and the AA2 family, which includes lignin-active class-II peroxidases. These enzymes alongside glycoside-hydrolases work in synergism to degrade lignocellulose (Figure 3). Additionally, other families show a correlation with oxidation or reduction of lignin-derived compounds such as the AA4 family, which includes vanillyl-alcohol oxidases; the AA5 family, which contains glyoxal oxidases/alcohol oxidases; and the AA6 family, which includes 1,4-benzoquinone reductases. Families AA3 and AA7 also show members potentially involved in lignin degradation and modification through Fenton reactions [43].

The basic mechanism of bacterial lignin deconstruction is analogous to that previously described for filamentous fungi, in which enzymes with oxidative activity present a central role. Enzymes are commonly described as being involved in lignin degradation in fungi such as laccases (E.C 1.10.3.2) and lignin peroxidases (E.C 1.11.1.7), and manganese peroxidases (E.C 1.11.1.13) are also present in bacteria [44,45]. Following Cazy database categorization, based on protein sequence and functional validation, most of the enzymes involved in lignin and aromatic compound oxidation are grouped in auxiliary activity (AA) families I and II. The AAI group comprises laccase/di-phenol:oxireductases/ferroxidases (EC 1.10.3.2), laccase-like multicopper oxidases (EC 1.10.3.-), and ferroxidases (EC 1.10.3.2). Group AA2 includes ascorbate peroxidases (EC 1.11.1.11), versatile peroxidases (EC 1.11.1.16), lignin peroxidases (EC 1.11.1.14), peroxidases (EC 1.11.1-), cytochrome-c peroxidases (EC 1.11.1.5), and manganese peroxidases (EC 1.11.1.13).

Laccases are multi-copper oxidases displaying activity against monophenols and compounds containing *p*-diphenol structures [46,47]. One of the first evidences of laccase activity produced by bacteria was observed for *Azospirillum lipoferum*, an alpha-proteobacteria obtained from rice rhizosphere. These enzymes displayed oxidative activity against *p*-phenylenediamine, hydroquinone, l-DOPA (l-3,4-dihydroxyphenylalanine), syringaldazine (4-hydroxy-3,5-dimethoxy-benzaldehyde azine), and ABTS (2,2′-azino-bis(3-ethylbenzothiazoline-6-sulfonic acid)) [48]. The enzyme responsible for the laccase activity was later purified and characterized as a multimeric protein. Similar findings have been described for fungal laccases, which can be monomeric, dimeric, or tetrameric [46].

Extremophile bacteria can also be laccase producers. Rezaei et al. (2017), purified a monomeric laccase (75 kDa) produced by the halotolerant bacterium *Aquisalibacillus elongatus* [49]. The purified enzyme showed activity against a variety of substrates including polyphenols such as tannic acid and monomeric phenols such as catechol, gallic acid, and guaicol [49]. In another report, Yu Chen et al. (2013) obtained a trimeric thermostable laccase from the thermophilic actinomycete *Thermobifida fusca*, produced using sugarcane biomass as the substrate. The purified enzyme had maximum activity at 60 °C, and exhibited oxidizing activity against the dyes p-phenylenediamine, and 2,6-dimethylphenylalanine [50].

Manganese peroxidases (MnP) contain a heme structure with a central manganese ion. These enzymes catalyze the reduction of substrates using H_2_O_2_ as an electron donor, catalyzing the oxidation of lignin [51,52]. Manganese peroxidases have been obtained from a great variety of bacteria. Oliveira et al. Oliveira (2009) reported the purification of MnPs from *Bacillus pumilus*, and *Paenibacillus* sp., isolated from wood-decaying material and paper mill effluent, respectively [51]. MnPs have also been identified in the alpha-proteobacteria *Aurantimonas manganoxydans* and *Erythrobacter* sp. [52]. An MnP produced by *Bacillus subtilis* from decomposing natural rubber in soil was reported by Nayanashree and Thippeswarny (2015) [53]. Although there are a number of reports on bacterial MnPs, they are much less characterized in comparison to laccases.

Lignin peroxidases are a widely studied class of proteins from the fungus *Phanerochaete chrysosporium*. Enzymes from this class can catalyze the oxidation of lignin and phenolic related compounds using peroxide [4]. Their counterpart in bacteria has been reported in a variety of genera including those typically found dwelling in lignin-rich environments. For example, a *Bacillus* sp. isolate (CSA105) from a paper mill effluent was able to produce classic plant cell wall degrading enzymes (e.g., cellulases) as well as lignin-active enzymes including a lignin peroxidase [54]. Still, regarding the phylum firmicutes, Gomare et al. (2008) reported the production and purification of a lignin peroxidase from *Brevibacillus laterosporus* MTCC 2298. The reported LiP displayed activity against the industrially-relevant dyes (i.e., methyl orange, and Blue-2B) [55].

Bacteria isolated from natural environments including actinobacteria have also been described as important players in lignin degradation mediated by LiP. Yee and Wood (1997) reported a LiP produced by *Streptomyces viridosporum* T7A with activity against 2,4-dichlorophenol, a compound usually found in pesticides and herbicides, which can be an important environmental contaminant [56]. Similar results were reported for a 49.7 kDa peroxidase from *Streptomyces* sp. AD001 that showed activity against 2,4-dichlorophenol [57].

In addition to the classical oxidases reported as presenting a central role in lignin degradation by bacteria, recent studies show other activities such as β-etherases enzymes, which are involved in lignin/phenolic compounds deconstruction/consumption. β-etherases cleave β-aryl ether bonds found in lignin and are receiving attention because they can degrade this kind of linkage in high-molecular-weight lignin. β-Etherase activity has been described for *Sphingomonas paucimobilis* SYK-6, and thus far it is one of the few enzymes with a non-oxidative mechanism against lignin [58].

One of the early studies on β-etherases was a report of the genes ligE and ligF coding for two β-etherases with similarities to the glutathione-*S*-transferase protein family in *Sphingomonas paucimobilis* SYK-6 [58]. Later, these β-etherases from *S. paucimobilis* were expressed in *E. coli* and shown to degrade high-molecular-weight lignin [58,59].

With the advent of new techniques to prospect new putative genes encoding lignin-modifying and deconstruction enzymes, and the development of more accurate protocols to identify products of lignin degradation and/or modification, it is expected that a more diversified group of proteins will be categorized as being involved in lignin biodegradation. Many reports of putative enzymes involved in lignin breakdown are based on the presence of proteins or sometimes gene expression when a microorganism is cultured in the presence of lignin or lignin-related compounds. However, more direct experimental data are still needed to validate the role of specific enzymes in lignin deconstruction. Examples include dioxygenases and veratryl alcohol oxidases, which do not have a widespread role in lignin and aromatic compound degradation in bacteria and require further validation of their role in lignin deconstruction.

## 4. Approaches to Access Bacterial Community Structure and Functionality

**Figure 4:**
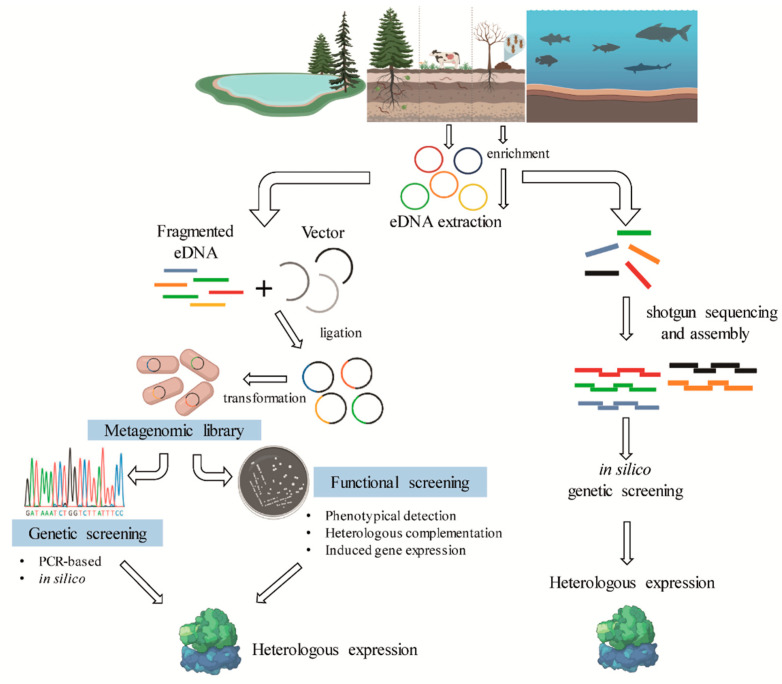
General scheme of the metagenomic strategies for the identification of lignin-degrading enzymes from different bacterial communities.

Metagenomics is a culture-independent approach that can be used to describe a microbiome in two different levels: structural and functional. At a structural level, a microbial community can be described regarding the microorganisms present, from phylum- to species-level, in addition, ecological interactions and evolutionary aspects can be explored [60,61]. In contrast, functional metagenomics explores the bacterial community genomic diversity of a sample, allowing the identification of genes and biochemical pathways prevalent in a microbiome (Figure 4). The choice of the target environment for omics studies is essential for mining biocatalysts. As shown in Table 1, in natural or modified microbiomes where lignin degradation occurs naturally, a repertoire of bacteria and genes that are involved in lignin degradation has been identified.

Different experimental approaches can be used to access the structural and functional profiles of microbial communities. Metataxonomics, for example, is based on the sequencing of phylogenetic marker genes such as 16S rRNA, providing information about the structural composition of a bacterial microbiome, sometimes reaching the hierarchical level of species [62,63,64]. On the other hand, shotgun metagenomics is an undirected sequencing of all the microbial DNA in a sample, allowing access to both the structural and functional community profile. Functional information can also be accessed through phenotypic screening, which includes the construction of metagenomic libraries, and the use of different techniques to identify genes with the desired function such as activity-based screening [65,66,67]. In addition to direct analysis of environmental samples using DNA sequencing techniques, culture-enrichment is a powerful tool to establish microbial consortia with desired properties. This technique basically consists of cultivating environmental communities on desired substrates such as lignin and/or lignocellulosic materials under specific conditions, thus providing the enrichment of microorganisms with the desired function. This selected microbial community is then subjected to the culture-independent methods previously described or the traditional culture-based ones.

In the next topic of this review, bacterial communities that use lignin as a carbon source and genes encoding lignin-modifying auxiliary activity (AAs) present in natural or enriched microbiomes will be described, with a special focus on omics techniques.

### 4.1. Microbiomes Characterized Using Metagenomic Approaches

#### 4.1.1. Soil

As previously discussed, environmental samples are an interesting source of microorganisms specialized in lignin deconstruction. One such example is soil bacteria, which play an active role in deconstructing the litter deposited in the Brazilian Caatinga soil. In a study that analyzed metagenomic libraries sequences from this microenvironment, considerable genetic potential for the deconstruction of lignin was observed in which members of the phyla Proteobacteria, Actinobacteria, and Acidobacteria acted mainly through enzymes belonging to auxifamilies AA3, AA7, and AA1 [68].

In the topsoil of coniferous forests, the phyla Proteobacteria, Acidobacteria, and Actinobacteria with the potential to encode genes for auxiliary enzyme activities, especially those belonging to AA3 families, have also been identified [69]. Interestingly, in another study, Wilhelm et al. (2019) [70], also characterized this microbiome in North America through the association of stable isotope probing (SIP), 16S rRNA gene amplicon, and shotgun metagenomics. The SIP technique was used to label microorganisms capable of assimilating the ^13^C-labeled substrates (hemicellulose, cellulose, or lignin) in the different soil layers. The authors demonstrated that bacterial deconstruction of lignin seems to occur throughout the entire soil column, and not just in the upper layer. Different bacteria with ligninolytic potential were identified both in the organic layer and in deeper mineral layers (Table 1). Among them are new lignin degraders from mineral soils belonging to the non-cultivable clades of Caulobacteraceae, Acidobacteria, Solirubrobacterales, Elusimicrobia, Nevskiales, and Cystobacteraceae. Likewise, the genes of the most abundant AA families (AA3, AA4, and AA6) were mainly from bacteria.

Antarctic soil microbial communities represent a genetic reservoir of cold-active ligninolytic enzymes. Bacteria belonging to the genera *Geodermatophilus*, *Thermobispora,* and *Amycolatopsis* exhibited potential for deconstructing lignin, with enzymes belonging to families AA7, AA3, and AA4, respectively, being the most prevalent [18].

Functional metagenomic analysis of a microbial community present in hydrocarbon-contaminated agricultural soils has shown that these environments also harbor bacteria specialized in lignin deconstruction. Members of the phylum Proteobacteria were identified as the main group, having genes for enzymes with auxiliary activity belonging to families AA3 and AA6 [71]. In these different microbiomes, bacteria belonging mostly to the phyla Proteobacteria, Acidobacteria, and Actinobacteria are central lignin decomposers. These bacteria use a different set of enzymes to deconstruct the lignin structure (Table 1), and it is worth mentioning that AA3 probably plays a role in lignin deconstruction in the soil as it is constantly part of the group of the most abundant enzymes.

#### 4.1.2. Invertebrate Digestive Tract

Bacterial communities inhabit a diversity of environments, and numerous bacteria live in association with invertebrate hosts such as termites, wood wasps, beetles, and wood-feeding roaches [78,79]. Insects play an important ecological role in the carbon cycle, and the breakdown of lignocellulosic fibers (e.g., wood, grass, and litter) [80]. Bacteria express and secrete carbohydrate-active enzymes in the invertebrate gut system, thus contributing to the digestion of lignocellulosic substrates [81].

To date, little is known about lignin degradation in the termite gut, whether it is performed by the termites themselves or mediated by unidentified gut bacterial communities involved in lignin degradation [82]. Although the majority of metagenomic studies of termite luminal fluid have shown no evidence for lignin degradation, some bacteria have been isolated and identified from termite gut such as *Trabulsiella* sp., *Rhodococcus erythropolis,* and *Streptomyces*, which have exhibited peroxidase and laccase activities [83,84,85].

Nevertheless, recent taxonomic and functional metagenomic analysis of gut microbiota of seven species of termites (subfamilies *Syntermitinae*, *Nasutitermitinae*, *Apicotermitinae*, and *Termitinae*) has revealed the presence of bacterial genes that encode enzymes that play a role in lignin deconstruction [72]. As a result, a set of CAZymes (CAZy database [86]), AA1, AA3, AA4, AA5, and AA6 families involved in the degradation and modification of aromatic compounds were detected in almost all termite species investigated. The most common bacterial genera harboring genes encoding for the AA1 family were *Legionella*, *Acinetobacter*, and *Pseudomonas*, for the AA5 family these were *Myxococcus* and *Streptomyces,* and for the AA7 family, it was *Actinoplanes*.

In another study of termites, Su et al. (2016), using 454 pyrosequencing of 16S rRNA, analyzed the microbiota gut of four species of termites (three species of wood-feeding termites and one species of a fungus-feeding termite) and *Spirochaetes* (11–55%), *Firmicutes* (7–18%), *Bacteroidetes* (7–31%), and *Proteobacteria* (8–14%) were detected as the main phyla for all four termites. Furthermore, based upon the automatically taxonomy-to-phenotype mapping, bacterial metabolic activities related to lignin degradation and modification of lignin-derived aromatic compounds were identified [87].

These findings show that the termite gut system contains bacteria whose genes encode enzymes for lignin degradation or modification, although their function in the termite gut remains unclear. Moreover, the bacterial communities differ between the midgut and hindgut segments (P1 to P5) of termites [88]. These differences may be related to oxygen availability. For instance, oxygen is low in the P3 segment, which may hinder oxidative degradation of lignin, while gut segments with aerobic conditions may harbor several species capable of lignin degradation [81].

In another study aiming to investigate the microbiota of the Asian long-horned beetle midgut, several bacterial reads with copper oxidase (Cu-oxidase) domains were searched in the protein family database (Pfam [89]), and many of these reads had a similarity to laccases, multicopper oxidases, and polyphenol oxidases. Additionally, a considerable number of annotated sequences had conserved domains with similarity to hypothetical proteins, which could represent uncharacterized laccase-type enzymes for lignin degradation. Furthermore, bacterial dye-decolorizing peroxidases that can cleave the β-aryl ether linkages in both syringyl and guaiacyl lignin in the presence of hydrogen-peroxide were detected. Genes for β-aryl ether degrading enzymes, classified as β-etherases or glutathione-*S*-transferases, were also identified [17].

A repertoire of lignocellulose-degrading enzymes has also been identified in the gut microbiome of the common black slug Arion. This study revealed more than 3383 CAZymes including multiple AAs families, associated with lignin degradation including members of the AA2, AA3, and AA4 families, which are involved in oxidative degradation, and the AA6 family, which catalyze the biodegradation of aromatic compounds such as monolignols [73].

Using shotgun sequencing and bioinformatics approaches, Agamennone et al. (2019) analyzed the gut microbiome associated with the soil invertebrate *Folsomia candida*. A total of 2004 genes encoding enzymes that degrade cellulose, starch, and lignin were detected. Among these, 1988 (99.2%) were of bacterial origin and mainly originated from *Proteobacteria* (43%) and *Actinobacteria* (36%). Among the CAZyme families, 81 genes were identified as auxiliary activities and the most abundant were the family members of AA3 family (32% of the total), AA6 (27%), and AA7 (17%). The repertoire of auxiliary activities identified suggested that the bacterial microbiome plays a role in the degradation or modification of lignin contributing to the adaptation of the invertebrate to life in the soil [74].

#### 4.1.3. Vertebrate Digestive Tract

Bacterial CAZymes are essential to ruminants as they hydrolyze fibrous plant materials, which are utilized by host animals as energy sources [90]. Studies of the ruminal microbiome at the domain level established that 97.5% of sequences belong to bacteria, 1.3% to archaea, and 0.9% to eukaryotes [75]. The phyla *Bacteroidetes*, *Firmicutes*, *Proteobacteria*, and *Fibrobacteres* were identified as being involved in plant biomass degradation [75,91,92].

According to Jose et al. (2017), approximately 0.46% of AA families of the total number of CAZyme families are involved in bovine rumen plant biomass deconstruction. It should be noted that AAs, which are involved in lignin degradation and act in conjunction with other hydrolytic enzymes, were observed with lower incidence (Table 1). Indeed, these results corroborate early reports that showed limited lignin degradation in the ruminal environment, mostly because of its natural microaerobic condition [93]. Five enzyme families essential for degradation or modification of lignin were detected in the rumen (i.e., AA6, AA5, AA4, AA7, and AA3). Phylogenetic analysis showed that species of bacteria belonging to genera *Prevotella*, *Bacteroides*, *Clostridium*, *Fibrobacter*, and *Ruminococcus* are key contributors of CAZymes inhabiting the cow’s rumen [75].

Beloqui et al. (2006) reported RL5, a gene coding for a polyphenol oxidase with laccase activity from a bovine rumen metagenomic library. Characterization of the recombinant laccase produced in *Escherichia coli* revealed its ability to oxidize different substrates such as syringaldazine, 2,6-dimethoxyphenol, veratryl alcohol, guaiacol, tetramethylbenzidine, 4-methoxybenzyl alcohol, 2,2′-azino-bis(3-ethylbenzothiazoline-6-sulfonic acid) (ABTS), and phenol red, over a wide pH range. Phylogenetic analysis assigned this laccase sequence to the genus *Bacteroides* [94].

Similarly, Ufarté et al. (2018) found in the bovine rumen microbiome producers of enzymes able to oxidize phenolic compounds such as ABTS (2,2′-azino-bis(3-ethylbenzothiazoline-6-sulfonic acid)), syringaldehyde, 1-hydroxybenzotriazole, acetosyringone, and 2,2′-azino-bis without the addition of mediators such as copper or manganese, presenting activity in a wide range of temperatures (45 to 60 °C) and pH (4.5 up to 5.5) [95].

Work focusing on the rumen microbiome of a more exotic ungulate, the camel, identified bacteria associated with lignin degradation. These bacteria were able to produce enzymes with auxiliary activities categorized into four families (AA3, AA4, AA6, and AA7), of which a 1,4-benzoquinone reductase belonging to AA6 accounted for >90% of the 1319 sequences. The auxiliary activity enzyme (AA6) was also abundant in the cow rumen biogas reactor microbiome [76]. Still, regarding the camel’s rumen microbiome, *Firmicutes* species contributed with 48% of the AAs found, *Bacteroidetes* species with 35%, *Spirochaetaes* with 8%, and *Fibrobacteres* and *Proteobacteria* species contributed with 4% [76].

Families of AAs represented 0.13% of the total number of CAZyme families found in the fecal samples of Asian elephants (Table 1). Among these, AA4 was the most abundant (50%) followed by AA6 members (20%) [77]. These numbers showed a different prevalence in comparison to the biogas fermenter, camel, and cow rumen where AA6 was the most abundant family [75,76].

### 4.2. Culture Enrichment

Culture enrichment using lignin as the main carbon source facilitates the identification of key microorganisms for its deconstruction and biotransformation. For example, the taxonomic and functional analysis of bacterial consortia derived from soil and chicken feces enriched in wood chips or filter paper, and subcultured in alkali lignin containing medium showed that *Pseudomonas* has a central role in the deconstruction of aromatic compounds. In addition to their high abundance in the consortia, they also had most of the genes involved in the *ortho*-cleavage and degradation pathways of benzoate and catechol. Other genera such as *Klebsiella*, *Variovorax*, *Leclercia*, and *Enterobacter* were also associated with lignin degradation [96].

Moraes et al. (2018) [97], used 16S rRNA gene sequencing and shotgun metagenomics to characterize a ligninolytic consortium obtained by cultivation of a sample of sugarcane soil in a medium containing low molecular weight soluble lignin as a carbon source. The authors reported an increase in the abundance of the families *Alcaligenaceae* and *Micrococcaceae* and reported for the first time the involvement of the genus *Pseudomaniobacter* in the degradation of aromatic compounds derived from lignin. Functional analysis of this consortium revealed the presence of different families of auxiliary activities (AA3, AA4, AA6, and AA7) and genes of the main pathways related to the degradation of aromatic compounds such as benzoate and phthalate (Table 2).

Three microbial consortia started from soils supplemented with wheat straw (WS1-M), switchgrass (SG-M), and corn stover (CS-M) were described as presenting a set of genes belonging to auxiliary activities families. AA6 family members were the most abundant, followed by members of the AA2, AA7, and AA4 families. The highest rate of lignin deconstruction, approximately 59%, was recorded for the SG-M consortium, which had a high abundance of the genera *Brevundimonas, Caulobacter*, *Pseudomonas*, *Citrobacter*, and *Aeromonas* [98].

In a microbial consortium generated from the cultivation of compost samples in media containing corn stover as the carbon source, 22 genes encoding AAs were assigned to Firmicutes and Proteobacteria. Only four genes encoding members of the AA2 family have been attributed to *Escherichia* and *Klebsiella* from the Proteobacteria phylum [99]. In contrast, a different straw-adapted compost microbial consortium was enriched for AA2 family genes, distributed among *Actinobacteria* species, a novelty, since these members were not previously recognized as active on lignin. Among the species identified were *Thermomonospora curvata*, *Mycobacterium xenopi*, *Amycolicicoccus subflavus*, and *Mycobacterium thermoresistibile* [100].

In a thermophilic consortium adapted to degrade switchgrass through multiple passages at 60 °C, several putative genes belonging to the AA1 family were assigned to *Thermus thermophilus*, *Sphaerobacter, Gemmatimonadetes spp.*, and *Paenibacillus spp*. Additionally, genes for families of glutathione S-transferases have also been found in the genus of *Hyphomicrobium*. This family of enzymes contains members that are involved in the cleavage of beta-aryl linked lignin dimers [101]. In another thermophilic bacterial consortium enriched with carboxymethylcellulose (CMC), six bacterial genomes were partially reconstructed and named as BZ1, BZ2, BZ3, BZ4, BZ5, and BZ6. Taxonomic and phylogenetic analysis suggest that three of the six reconstructed genomes belong to new bacteria: *Thermobacillus* (BZ1); a member of a new genus in the family *Paenibacillaceae* (BZ3), similar to *Paenibacillus* and *Cohnella*; and a member of a new deep-branching family in the *Clostridia* (BZ6) class. Six families of auxiliary activities related to the degradation of lignin (AA1, AA2, AA4, AA6, and AA7) were found in the six reconstructed genomes [102].

### 4.3. Metatranscriptomics and Metaproteomics Approaches

#### 4.3.1. Metatranscriptomics

**Figure 5:**
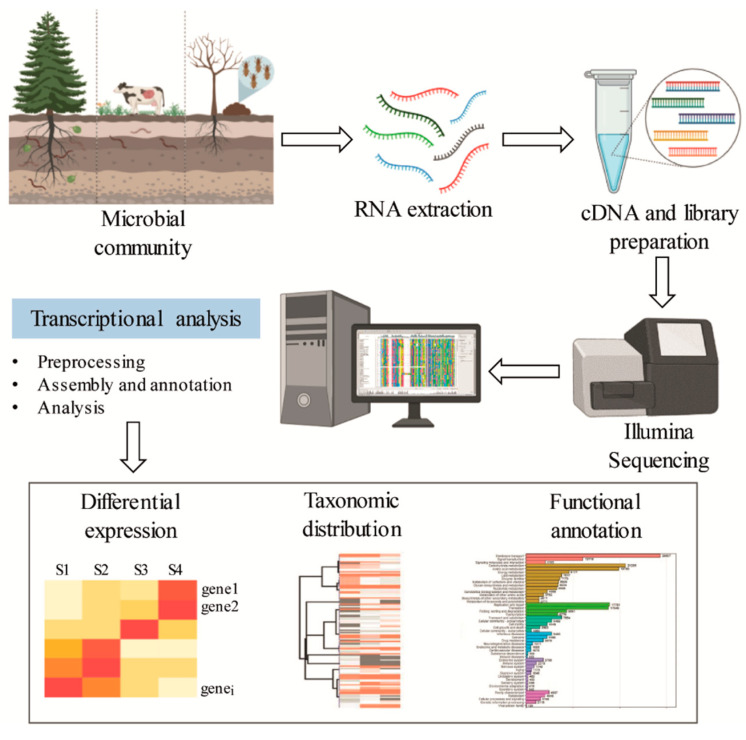
General scheme for functional and genetic profiling of oxidative enzymes exploiting metatranscriptomic sequence data.

Metatranscriptomics is the study of the rRNA and mRNA of a microbial community, which provides information on the active functional profile through the evaluation of gene expression (Figure 5) [103]. In contrast to metagenomics, which describes the composition, function, and relative abundance of different members of a microbial community, metatranscriptomics provides insight into the metabolically active part of the community. Therefore, metatranscriptomic analysis is restricted to transcribed genes, and the non-transcribed portions of the genome that are frequent in metagenomic data are not present [104]. However, technical limitations for effective RNA recovery, low quantities of bacterial mRNA in samples, and the short lifetime of mRNA can restrict the application of this approach. The transcriptional profile of auxiliary activity genes involved in lignin degradation is summarized in Table 3.

Active metabolic pathways of the microbial community in a thermophilic composting operation in the São Paulo Zoo Park have been unraveled through metatranscriptomics [105]. Lignin deconstruction occurred sequentially and synergistically by the action of ligninases that reached peak abundance at the end of the composting process. The majority of ligninolytic enzymes belonging to the classes AA2 (7–28% of all AAs) and AA6 (32–66%), followed by moderate amounts of AA3, 4, and 7, were found in all samples. Members of *Clostridiales*, *Bacillales*, and *Actinomycetales* were implicated as the major compost-degrading microbes, particularly in the thermophilic and mature stages of the composting process [105].

The temporal expression dynamics of a set of plant biomass-degrading enzymes by a bacterial consortium growing on sugarcane bagasse was studied. Here, Jiménez et al. (2018) described the importance of the expression of auxiliary activities AA2 and AA6. The highest expression levels of transcripts encoding AA2 and AA6 members of families derived from *Paenibacillus* and *Brevundimonas* were observed at early stages (at 12 h) of the consortium’s growth. According to the authors, this could be related to cell protection against oxidative damage and electron transfer. Finally, the expression of catalase-peroxidases (AA2) was carried out by *Chryseobacterium* and *Brevundimonas* at the final stages (192 h), suggesting that the degradation of lignin occurred in the final stages of growth of the consortium [106].

Moreover, Žifčáková et al. (2017) investigated the glycoside hydrolases and auxiliary enzyme contribution of fungi and bacteria in two different stations on top forest soil. A high diversity of transcription in families of AA3 (mixed activities in lignocellulose) and AA1 (laccases) was reported. Concerning CAZymes functional groups, those destined for cellulose were more transcribed, followed by those that act on lignin [69].

Tokuda et al. (2018) identified transcripts encoding three families of auxiliary activities belonging to AA6, AA4, and AA2 involved in bioconversion of phenolic compounds in the hindgut compartment from termites (*Nasutitermes takasagoensis*) [107]. Additionally, Marynowska et al. (2017), studied the lignocellulolytic potential of the higher termite gut prokaryotes. They reported transcripts encoding enzymes such as AA4 (vanillyl alcohol oxidase), and AA6 families (1,4-benzoquinone reductase) [108].

#### 4.3.2. Metaproteomics

**Figure 6:**
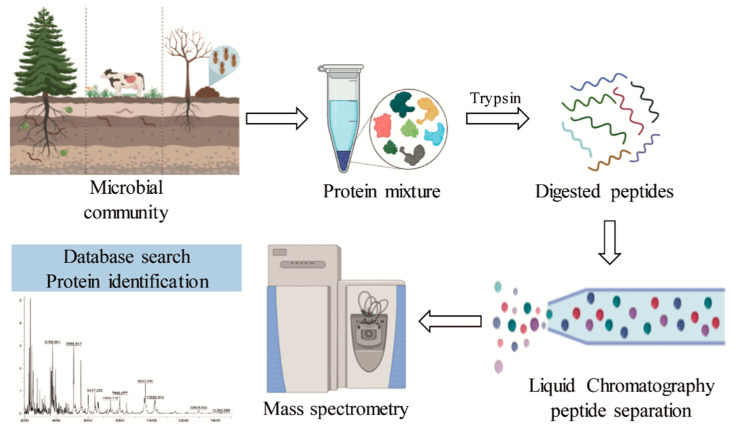
General scheme of the metaproteomic analysis for the identification of lignin-degrading enzymes from different bacterial communities.

Metaproteomics allows the assessment of the complete protein content of an environmental microbiota (Figure 6). The main challenges of metaproteomics include sample complexity, low peptide identification, and lack of complete databases [110,111]. Although there are still only a few studies that have reported the presence of enzymes related to lignin degradation, the use of this approach in lignocellulolytic bacterial communities has demonstrated the presence of auxiliary activities involved in the deconstruction of lignin (Table 3).

For example, the analysis of the bacterial consortium secretome adapted to using rice straw as a carbon source showed the presence of 16 domains of auxiliary activities belonging to three families (AA2, AA7, and AA10). Interestingly, in addition to single-domain CAZymes, other proteins with multiple domains have been identified, for example, ligninolytic proteins containing three distinct AA2 domains. The main groups responsible for the deconstruction of this biomass belonged to the phyla Proteobacteria and Bacteroidetes [109].

In contrast, for another consortium adapted to corn stover, only one AA2 protein (catalase/peroxidase) from *Escherichia coli* was considered potentially involved in the degradation of lignin [99]. Likewise, in another bacterial consortium adapted for using switchgrass as a carbon source, only a putative laccase assigned to the *Gemmatimonadetes* was identified [101].

## 5. Application of Lignin-Active Enzymes

Nowadays, with a growing interest in green processes, microbial enzymes are the major biological players sought after for industrial applications. Lignin-active enzymes are prospected for many applications such as decontamination of industrial dyes [112], general delignification for diminishing lignocellulose recalcitrance [16], and the production of aromatic compounds of industrial relevance [8]. As previously discussed, enzymes produced by filamentous fungi are the prime source for industrial applications, however, bacteria can produce enzymes with interesting kinetic parameters that should be further exploited.

The major challenge in using enzymes produced by bacteria instead of those secreted by classic filamentous fungi such as *Trichoderma* sp. is related to enzyme production levels. Filamentous fungi that are prospected for enzyme production are naturally specialized in secreting enzymes with activity against lignin and holocellulose [113,114]. The utilization of similar enzymes obtained from bacteria requires recombinant production of these enzymes in host cells suitable to industrial conditions. In this regard, heterologous expression and a direct evolution approach to improve catalytic activity, enhance expression levels, and improve laccase stability have been pursued to convert bacterial laccases into highly efficient and commercially valuable biocatalysts [13].

Bio-delignification by AA1 family (laccases) provides a clean and efficient treatment method of lignocellulose without damaging the cellulose, which is important for commercial bioethanol production. Researchers have shown that laccases can work more efficiently in combination with hemicellulases for lignocellulose saccharification. However, Rocha-Martín et al. [115], reported that supplementation of laccase in enzymatic hydrolysis showed contradictory results, depending on pretreatment type and biomass used. The addition of laccase to softwood hydrolysis resulted in high glucose yield, while a negative effect was obtained for hardwood hydrolysis.

AA1 families have successfully reported effectiveness for kraft pulps and biobleaching. Arias et al. (2003) showed that the biobleaching of eucalyptus kraft pulps by lacasse (AA1_1 family) from *Streptomyces cyaneus* CECT 3335 using ABTS as a mediator resulted in the significant decrease in the kappa number up to 2.3 U and increased pulp brightness. An alkaline and halotolerant bacterial laccase (*SilA*) produced by *Streptomyces ipomoea* CECT 3341 was also used to enhance the conventional chemical bleaching process of an industrial eucalyptus kraft pulp [47,116].

Lignin deconstruction and detoxification of aromatic compounds by bacterial enzymes is a widely investigated topic. However, details on bacterial uptake and metabolization of the compounds released during lignin breakdown is still a relatively less investigated topic. Recent studies are not only focusing on how the bacterial enzymatic machinery works in lignin deconstruction, but also on investigating how these compounds are metabolized. Although the intention of this review is not to describe the detailed pathways, it is clear that the bacterial metabolism of lignin can generate interesting biotechnological products such as bioplastics, vanillin, and lipids that can be further transformed into biofuels [43].

## 6. Conclusions and Future Perspectives

The use of metagenomic approaches to study lignocellulosic microbial communities present in different environments has revealed the ligninolytic potential of several bacteria including new non-cultivable clades such as *Caulobacteraceae*, *Solirubrobacterales,* and *Cystobacteraceae*. It has also allowed access to a set of potential bacterial genes for auxiliary activities, belonging mainly to families AA2, AA3, AA4, AA5, AA6, and AA7, which are involved in the degradation of lignin and other components of the plant cell wall. One limitation of metagenomics is that it does not provide information about active microbial communities. This is why complementary approaches such as metatranscriptomics and metaproteomics need to be used. Studies using these two latter approaches have shown the presence of auxiliary bacterial activities related to lignin degradation (Table 3). To date, there are still few studies related to bacterial degradation of lignin using omics approaches, and these mainly use metaproteomics. Future work aimed at the bacterial degradation of lignin needs to take advantage of the integrative use of the three omics approaches. Together, these will likely generate a more comprehensive understanding of microbial degradation and/or modification of lignin.

## Figures and Tables

**Table 1:** Auxiliary activity families (AAs) present in different bacterial communities as surveyed from metaomics studies.

DNA Source	Bacteria Associated with Lignin Degradation	AAs Families	References
Brazilian Caatinga soil	*Proteobacteria, Actinobacteria, Acidobacteria*	AA1, AA3, AA7	[68]
Forest soil	*Proteobacteria*, *Acidobacteria*, and *Actinobacteria*	AA3	[69]
Soil	*Caulobacteraceae, Acidobacteria, Solirubrobacterales, Elusimicrobia, Nevskiales*, and *Cystobacteraceae*	AA1, AA3, AA4, AA5, AA6, and AA7	[70]
Antarctic soil	*Geodermatophilus*, *Thermobispora*, and *Amycolatopsis*	AA3, AA4, and AA7	[18]
Agricultural soil	*Proteobacteria*	AA3, and AA6	[71]
Termite gut microbiome	*Legionella, Acinetobacter*, and *Pseudomonas, Myxococcus, Streptomyces,* and *Actinoplanes*	AA1, AA3, AA4, AA5, and AA6	[72]
Arion gut microbiome	n.s.	AA2, AA3, AA4, and AA6	[73]
*Folsomia* gut microbiome	*Proteobacteria* and *Actinobacteria*	AA3, AA6, and AA7	[74]
Bovine rumen	*Prevotella, Bacteroides, Clostridium, Fibrobacter,* and *Ruminococcus*	AA6, AA5, AA4, AA7, and AA3	[75]
Camel rumen	*Firmicutes, Bacteroidetes, Spirochaetaes, Fibrobacteres,* and *Proteobacteria*	AA3, AA4, AA6, and AA7	[76]
Elephant feces	n.s.	AA4 and AA6	[77]

n.s. = data not shown.

**Table 2:** Auxiliary activity families (AAs) and metabolic pathways of aromatic compounds consumption present in different bacterial consortium identified in metagenomic studies.

Consortium Source and Bacteria with Ligninolytic Potential	Substrate	AA Families	Pathways of Consuming Aromatic Compounds	References
Enrichment from chicken feces and soil (*Pseudomonas*, *Klebsiella*, *Variovorax*, *Leclercia*, and *Enterobacter*)	Alkali lignin	-	Catechol *ortho*- cleavage and benzoate degradation pathways	[96]
Enrichment from sugarcane plantation soil (Proteobacteria, Actinobacteria, Firmicutes, *Alcaligenaceae*, and *Micrococcaceae*)	Low-molecular- weight (LW) lignin	AA2, AA3, AA4, AA6, and AA7	Benzoate degradation to catechol, catechol *ortho*-cleavage, catechol meta- cleavage, and phthalate degradation	[97]
Enrichment from soil (*Brevundimonas*, *Caulobacter*, *Pseudomonas*, *Citrobacter*, and *Aeromonas*)	Wheat straw switchgrass and corn stover	AA2, AA4, AA6, and AA7	-	[98]
Enrichment from compost ecosystems (*Proteobacteria* and Firmicutes)	Corn stover	AA2, AA3, AA4, AA6 and AA7	-	[99]
Enrichment from compost ecosystems (*Symbiobacterium* *thermophilum, T.* *curvata*, *Mycobacterium* *xenopi*, *Amycolicicoccus* *subflavus* and *Mycobacterium* *thermoresistibile*)	Rice straw	AA2	-	[100]
Enrichment from compost ecosystems (*Sphaerobacter*, *Hyphomicrobium*, *Thermus* *thermophilus*, *Sphaerobacter*, *Gemmatimonadetes*, *Paenibacillus*)	Switchgrass	AA2	*Ortho*-cleavage of protocatechuate and 4- hydroxyphenylacetate degradation	[101]
Enriched from compost ecosystems (*Thermobacillus* species, *Bacillus*)	CMC	AA1, AA2, AA4, AA6, and AA7	-	[102]

**Table 3:** Families of auxiliary activities (AAs) present in different bacterial communities, as researched from metaomics studies.

Sample	Omics Techniques Applied	AAs Families	References
Compost ecosystems	Metatranscriptomics	AA2, AA3, AA4, AA6, and AA7	[105]
Soil microbiota	Metatranscriptomics	AA2 and AA6	[106]
Forest soil	Metatranscriptomics	AA1 and AA3	[69]
Termite gut	Metatranscriptomics	AA2, AA4, and AA6	[107]
Termite gut	Metatranscriptomics	AA4 and AA6	[108]
Bacterial consortium	Metaproteomics	AA2 and AA7	[109]
Bacterial consortium	Metaproteomics	AA2	[99]
